# Mitochondrial Targeting of MVA Pathway Triggers Severe Inhibition of Post-Squalene Cholesterol Biosynthesis: Transcriptomic and Proteomic Insights in Yeast

**DOI:** 10.3390/molecules31121999

**Published:** 2026-06-07

**Authors:** Nan Tang, Yuliang Xu, Changfu Li, Yansheng Zhang

**Affiliations:** Shanghai Key Laboratory of Bio-Energy Crops, School of Life Sciences, Shanghai University, Shanghai 200444, China; nantang@shu.edu.cn (N.T.); xyltta0ssaw@shu.edu.cn (Y.X.); changfuli@shu.edu.cn (C.L.)

**Keywords:** mitochondria, MVA, post-squalene pathway, squalene

## Abstract

Expression of the mevalonate (MVA) pathway in yeast mitochondria is deployed at commercial scale for production of squalene, because mitochondria contain abundant acetyl-CoA, the starting molecule of the MVA pathway. However, it is still unknown whether this strategy is effective in boosting the post-squalene pathway. Here the potential of this strategy is explored for production of the post-squalene chemical cholesterol, a precursor of many valuable steroidal drugs. A cholesterol-producing yeast strain, named CEN-Cho, was constructed by expressing the biosynthetic genes leading to cholesterol, accompanied by the augmentation of the cytosolic MVA pathway. The CEN-Cho strain produced 60.17 ng/mg fresh weight (FW) of squalene and 121.75 ng/mg FW of cholesterol in shake flask cultivation. When the complete MVA pathway was introduced into the mitochondria of CEN-Cho, yielding CEN-Cho-mMVA, the squalene level was increased to 325.24 ng/mg FW. Unexpectedly, the yield of cholesterol produced by CEN-Cho-mMVA was decreased to 1.5 ng/mg FW, demonstrating significant suppression on the post-squalene pathway due to the mitochondrial engineering. Comparative transcriptomic and proteomic analyses of the engineered cells provide insights into the metabolic and regulatory bottlenecks underlying this inhibition. This work reveals that the introduction of the MVA pathway into mitochondria generally inhibits the post-squalene pathway in yeast.

## 1. Introduction

As a central precursor of many steroid-type drugs, cholesterol plays indispensable physiological roles in living organisms and holds broad industrial value. For example, the tetracyclic sterane backbone of cholesterol can undergo oxidative modifications, ring cleavage, and other structural transformations to yield diverse valuable derivatives, such as vitamin D [1], bile acids [2], and steroid hormones including sex hormones [3]. In recent years, cholesterol and its derivatives have been widely applied in clinical practice. For instance, in the nervous system, desmosterol (a derivative of cholesterol) acts as a ligand to activate liver X receptors (LXRs), regulating myelin regeneration and inflammatory responses, thereby offering a promising therapeutic target for neuro-degenerative diseases [4,5].

**Figure 1:**
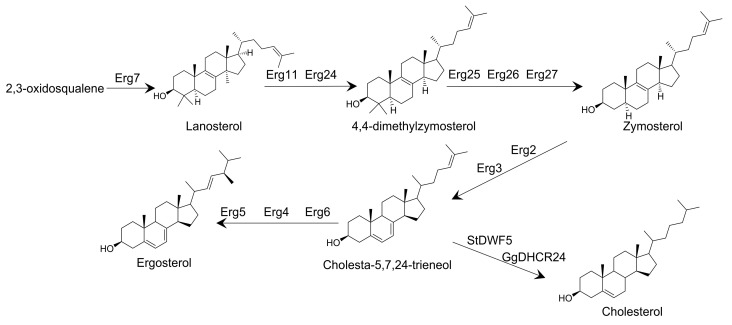
Biosynthetic steps for cholesterol biosynthesis in *S. cerevisiae*.

Cholesterol is biosynthesized via the post-squalene pathway. In yeast, the biosynthetic pathway for cholesterol is as follows (Figure 1): 2,3-Oxidosqualene is first cyclized to lanosterol by lanosterol synthase (Erg7), and lanosterol is subsequently converted into cholesta-5,7,24-trieneol, through the sequential action of Erg11, Erg24, Erg27, Erg26, Erg25, Erg2 and Erg3. At the final stage, two heterologous enzymes (DHCR7 and DHCR24) convert cholesta-5,7,24-trieneol into cholesterol. The production of 184.7 mg/L of cholesterol in *Saccharomyces cerevisiae* was achieved by expressing *StDWF5* (encoding DHCR7) from *Solanum tuberosum* and *GgDHCR24* (encoding DHCR24) from *Gallus gallus* [6]. Under the activities of three endogenous enzymes (Erg6, Erg4 and Erg5), cholesta-5,7,24-trieneol is converted into ergosterol (Figure 1). Accordingly, inducible suppression of *Erg6* expression has been commonly employed as a metabolic engineering strategy to enhance cholesterol production in yeast by restricting carbon flux into ergosterol biosynthesis [6,7]. In eukaryotic systems, sterol biosynthesis is tightly controlled by subcellular compartmentalization and homeostatic regulation. In yeast, all post-squalene sterol enzymes are predominantly localized to the endoplasmic reticulum (ER), and the transcription factor Upc2 acts as a master regulator to coordinate sterol biosynthetic gene expression [8,9]. In mammalian cells, the rate-limiting enzyme HMG-CoA reductase is also ER-anchored, and pathway activity is feedback-regulated by sterol levels via SREBP signaling [10]. In yeast cells, lipid droplets serve as major sterol storage compartments, while mitochondria provide a rich acetyl-CoA pool but are not inherently equipped to mediate post-squalene sterol metabolism [11].

Mitochondria offer new opportunities for metabolic engineering, as they contain abundant acetyl-CoA [12], and compartmentalization of the engineered pathway in mitochondria could also alleviate regulatory constraints imposed by cytosolic metabolism [13]. For instance, Yee et al. reported that mitochondrial engineering increased geraniol production to levels that were six-fold higher than those achieved through the corresponding cytosolic engineering in yeast [14]. The integration of both mitochondrial and cytosolic engineering in *S. cerevisiae* elevated isoprene titers to 2.5 g/L, representing 1.6-fold and 2.1-fold improvements relative to cytosolic and mitochondrial engineering alone, respectively [15]. Similarly, Zhu et al. employed a combined strategy to boost squalene production to 21.1 g/L [16].

Although mitochondrial engineering has demonstrated substantial potential in natural product biosynthesis, its application has thus far been largely confined to squalene and its upstream metabolites [16]. Whether this strategy can be effectively extended to boost the post-squalene pathway remains unresolved. This question is particularly critical because many steroidal drugs are derived from the post-squalene pathway [17,18,19].

In this study, we systematically evaluated the impact of mitochondrial engineering on the biosynthesis of squalene and several post-squalene products. Consistent with previous reports [16], mitochondrial engineering significantly enhanced squalene accumulation in our engineered yeast cells. However, for post-squalene products, only the production of lanosterol was improved, whereas significant suppression was observed for biosynthesis of both cholesterol and ergosterol. Transcriptomic and proteomic profiling on the engineered yeast cells provided insights into possible mechanisms underlying this metabolic bottleneck.

## 2. Results

### 2.1. Construction of a Cholesterol-Producing Yeast Strain

Production of cholesterol in yeast can be achieved by heterologous expression of 7-dehydrocholesterol reductase (DWF5) and 24-dehydrocholesterol reductase (DHCR24) (Figure 1). Thus, expression cassettes of *StDWF5* from *S. tuberosum* and *GgDHCR24* from *G. gallus* [20] were integrated into the genome of CEN.PK2-1C. To enhance the precursor supply, squalene synthase (hSQS) and squalene epoxidase (hSE) from *H. sapiens* and acetyl-CoA synthetase 1/2 (Acs1/2), farnesyl diphosphate synthase (Erg20), acetyl-CoA thiolase (Erg10) and the truncated HMG-CoA reductase mutant (tHmg1) from *S. cerevisiae* were all overexpressed via genome integration. To down-regulate *Erg6* expression, an antisense RNA strategy was employed, in which a reverse-oriented *Erg6* transcript was placed under the control of the GAL1 promoter. The resulting yeast strain was designated CEN-Cho (Table S3).

**Figure 2:**
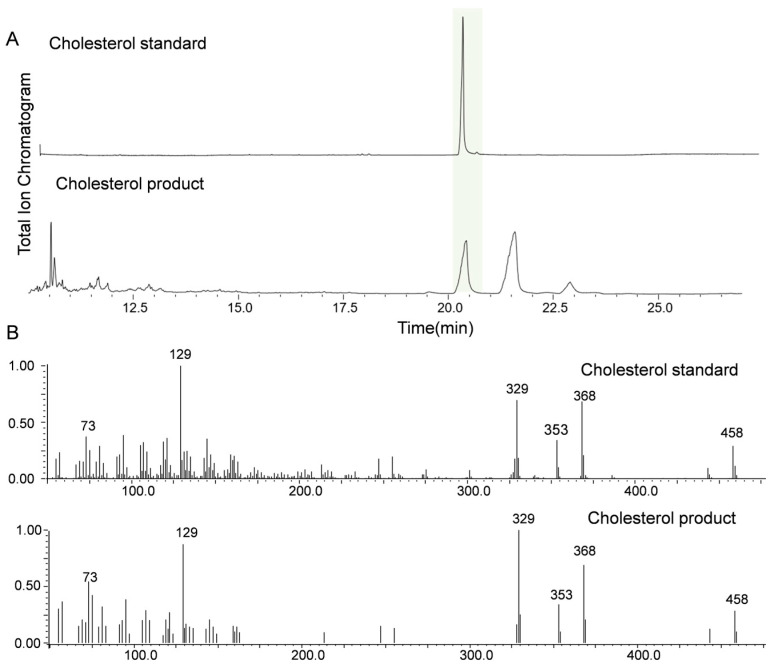
GC-MS analysis of the cholesterol produced by CEN-Cho strain. Total ion chromatograms (TICs) (**A**) and mass fragmentation spectrum (**B**) are shown for the cholesterol product along with its chemical standard. The green-shaded region in the chromatogram denotes the cholesterol product peak corresponding to its chemical standard.

As shown in Figure 2, the CEN-Cho strain was able to produce a compound with identical retention time and the same mass spectrum as cholesterol standard (Figure 2A,B). Fermentation in a 120 h flask culture led to the production of 0.40 µg/mg FW of cholesterol. Compared with that (3.10 µg/mg FW) in the parent strain CEN.PK2-1C, the content of ergosterol (0.20 µg/mg FW) in the CEN-Cho strain was decreased by over 90%, indicating that the heterologous expression of *StDWF5* and *GgDHCR24* effectively redirected metabolic flux away from the ergosterol pathway. Interestingly, the sum of cholesterol and ergosterol (cholesterol: 0.40 µg/mg FW; ergosterol: 0.20 µg/mg FW) in CEN-Cho was much less than the content of ergosterol alone (3.10 µg/mg FW) in the parent strain CEN.PK2-1C. The synthesis of exogenous cholesterol may remodel the membrane sterol profile of CEN-Cho [21], which further triggers endogenous sterol homeostatic regulation and consequently leads to the global repression of carbon flux throughout the sterol biosynthetic pathway.

### 2.2. Mitochondrial Targeting of MVA Pathway Triggers Severe Inhibition of Post-Squalene Pathway

**Figure 3:**
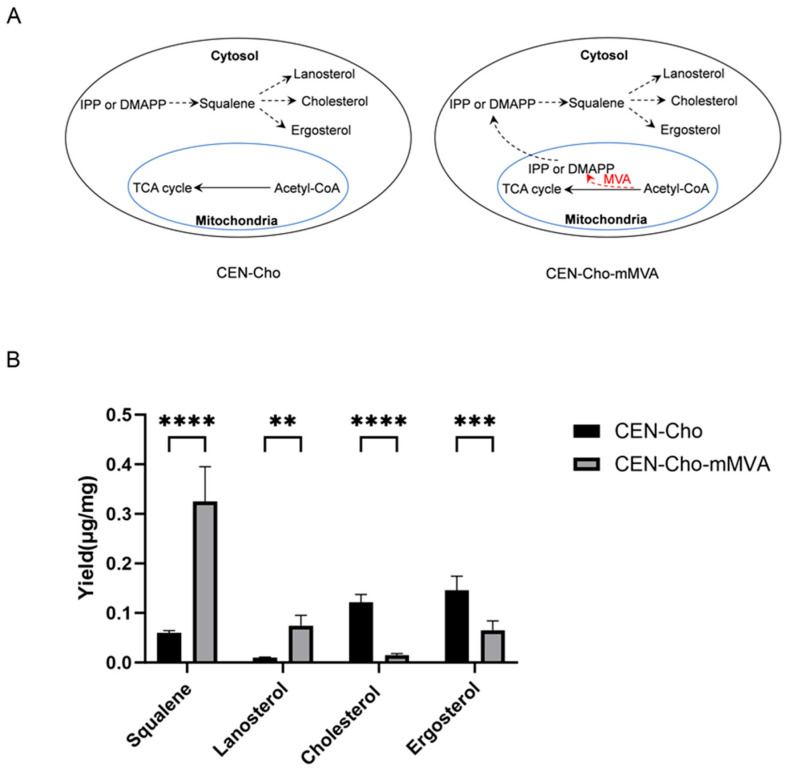
The effect of the mitochondrial MVA pathway engineering on the biosynthesis of squalene and post-squalene compounds (including lanosterol, cholesterol and ergosterol) in yeast. (**A**) Schematic diagram of the engineering strategy for CEN-Cho and CEN-Cho-mMVA. Based on the cholesterol-producing strain CEN-Cho, the complete MVA pathway, consisting of Erg10, Erg13, tHmg1, Erg12, Erg8 and Erg19, was targeted to its mitochondria, yielding a yeast strain named CEN-Cho-mMVA. (**B**) The two strains, CEN-Cho and CEN-Cho-mMVA, were compared in their abilities to synthesize squalene and three post-squalene compounds (lanosterol, cholesterol and ergosterol). Error bars represent standard deviations from biological triplicates. ** stands for *p* < 0.01, *** stands for *p* < 0.001 and **** stands for *p* < 0.0001, representing significant differences the CEN-Cho and CEN-Cho-mMVA groups. The red-labeled MVA indicates the expression of the entire MVA pathway in mitochondria.

Previously, Zhu et al. reported that engineering the MVA pathway in mitochondria could substantially increase squalene yield in yeast cells [16]. To investigate whether this engineering strategy is also effective in improving biosynthesis of post-squalene metabolites, such as cholesterol, the complete MVA pathway, consisting of Erg10, Erg13, tHmg1, Erg12, Erg8 and Erg19, was targeted to the mitochondria of the CEN-Cho strain (Figure 3A), generating a yeast strain named CEN-Cho-mMVA. Consistent with the previous report [16], expression of the complete MVA pathway in mitochondria largely increased squalene yield, with a 440.5% improvement found in CEN-Cho-mMVA relative to CEN-Cho (Figure 3B). However, among the three post-squalene compounds (lanosterol, cholesterol and ergosterol) measured in this study, only lanosterol showed an increase following the mitochondrial engineering, whereas the contents of cholesterol and ergosterol were decreased by 87.7% and 55.1%, respectively (Figure 3B). CEN-Cho-mMVA displayed impaired growth compared with the CEN-Cho strain (Figure S1), when cultured in an induction medium containing 2% galactose. Given that ergosterol is indispensable for maintaining fungal membrane integrity and normal physiological functions, the pronounced decline in endogenous ergosterol biosynthesis is highly likely to account for the growth retardation phenotype observed in CEN-Cho-mMVA.

### 2.3. Changes in mRNA and Protein Levels in Response to Mitochondrial MVA Pathway Targeting

To gain insights into the mechanism underlying the adverse effect of mitochondrial MVA pathway engineering on the post-squalene pathway, transcriptomics and MS-based proteomics were utilized to monitor changes in transcript and protein abundances between CEN-Cho and CEN-Cho-mMVA. The transcriptomic sequencing data of all samples showed high quality, with consistent sequencing depth and high mapping rates to the reference genome (Table S1), confirming the reliability of the transcriptomic data for subsequent analyses. Principal component analysis (PCA) showed a clear separation of the two strains, with CEN-Cho and CEN-Cho-mMVA forming distinct clusters, while replicates within each treatment exhibited high similarity (Figures S2 and S3). A total of 2182 differentially expressed genes (DEGs) and 702 differentially expressed proteins (DEPs) were identified in the comparison group. Enrichment analysis showed that the DEGs were mainly enriched in the processes of spliceosome, pyruvate metabolism, oxidative phosphorylation, citrate cycle, and glycolysis (Figure S4) and the DEPs in several metabolic pathways, such as those for biosynthesis of secondary metabolites, steroids, and glycerolipids (Figure S5).

**Figure 4:**
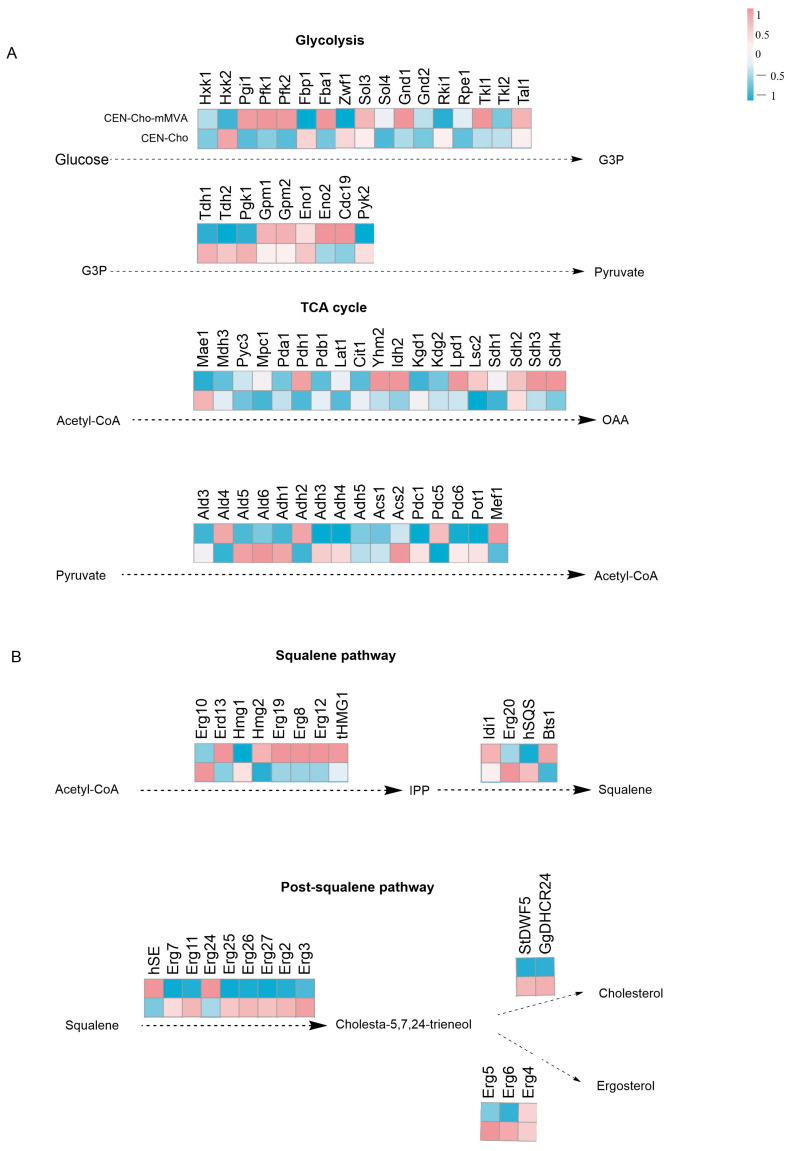
The effect of the mitochondrial targeting of the MVA pathway on the expression of enzymes involved in central carbon metabolism (glycolysis and TCA cycle) (**A**), squalene biosynthesis and post-squalene metabolic modules (**B**). The heatmap was drawn using R software (version 4.3.3). The scale from −1 (blue) to +1 (red) represents increasing in protein expression.

Next, we narrowed the analysis of DEGs/DEPs to three core metabolic modules: central metabolism, squalene biosynthesis, and post-squalene metabolism. The central metabolism module included cytosolic glycolysis, cytosolic acetyl-CoA supply, and the mitochondrial tricarboxylic acid (TCA) cycle. As shown by the transcriptome (Figure S6), the mitochondrial-integrated MVA pathway genes, including *MLS-Erg19*, *MLS-Erg8*, *MLS-Erg12*, *MLS-tHmg1*, *MLS-Erg13* and *MLS-Erg10*, were all highly expressed in the CEN-Cho-mMVA strain, confirming that they were successfully transcribed. Interestingly, integration of the MVA pathway in mitochondria decreased the expression of most of the cytosolic transcripts for squalene biosynthesis, such as *Hmg2*, *Erg12*, *Erg8*, *Idi1*, *Erg20*, *hSQS* and *Bts1* (Figure S6). Conversely, the protein levels of most of the squalene pathway enzymes were significantly up-regulated in CEN-Cho-mMVA compared with CEN-Cho (Figure 4), which could account for the increased levels of squalene found in CEN-Cho-mMVA (Figure 3). Overall, the post-squalene pathway genes showed the same trend in the changes between the transcriptomic and proteomic analyses (Figure S6 and Figure 4), implying that the change in the post-squalene module was mainly due to transcriptional regulation. In the post-squalene module, SE is the first critical enzyme converting squalene into lanosterol [11]. The expression level of *hSE* was significantly up-regulated, whereas the genes involved in the steps beyond lanosterol towards either ergosterol or cholesterol, except for *Erg24*, were all significantly down-regulated in CEN-Cho-mMVA (Figure S6), which agreed with the observation that more lanosterol but markedly less ergosterol and cholesterol were produced in CEN-Cho-mMVA, when compared with CEN-Cho (Figure 3).

For the glycolysis process, the upper part of the pathway, that is, the process of converting glucose into glyceraldehyde-3-phosphate (G3P), was generally up-regulated after mitochondrial MVA pathway engineering, which can be seen at both transcript and protein levels (Figure S6 and Figure 4). In contrast, the change trend between the transcripts and proteins in the downstream pathway of glycolysis, which converts G3P to pyruvate, were not consistent or even opposite (Figure S6 and Figure 4), suggesting that post-transcriptional regulation largely occurs in glycolysis. At the protein level, most of the enzymes in the downstream steps up to pyruvate were down-regulated (Figure 4), which in turn would lead to decreased accumulation of cytosolic pyruvate. In yeast, pyruvate can be fluxed into the biosynthesis of acetyl-CoA in the cytoplasm or into the TCA cycle in mitochondria [22]. With respect to the processes of cytosolic acetyl-CoA supply and the mitochondria TCA cycle, the transcriptome and proteome analysis data were overall correlated. Generally, the TCA cycle was strengthened, whereas the cytosolic acetyl-CoA flux was attenuated (Figure S6 and Figure 4).

## 3. Discussion

Many steroidal drugs, such as diosgenin and ursodeoxycholic acid [23,24], come from the post-squalene pathway. For this reason, there is growing interest in production of steroidal compounds of interest through the endogenous post-squalene pathway of microbial hosts [6,24]. For synthesis of steroidal compounds in *S. cerevisiae*, one effective method is to utilize subcellular organelles, such as lipid bodies [19,25,26]. Mitochondria have also been widely explored in metabolic engineering [14,15,16], as they contain a much higher concentration of acetyl-CoA than the cytosol [12,13]. When a complete MVA pathway was integrated in mitochondria of *S. cerevisiae*, the resulting strain could produce 21.1 g/L squalene [16]. Inspired by that report, this study was conducted to investigate whether mitochondrial MVA engineering can also be beneficial for synthesis of post-squalene chemicals, utilizing cholesterol as an example in this case.

Consistent with the previous study [16], we did observe a 5.4-fold increase in squalene yield (Figure 3) when the MVA pathway was targeted to mitochondria. Presumably, the increase in squalene might have primarily resulted from utilization of the mitochondrial acetyl-CoA pool by the relocated MVA pathway, as most of the transcripts for cytosolic acetyl-CoA biosynthesis (*Acs1* and *Acs2*) and the cytosolic MVA pathway (*Erg8*, *Erg12*, *Erg13*, and *Erg19*) showed decreased expression in CEN-Cho-mMVA relative to CEN-Cho (Figure S6). Unexpectedly, engineering the MVA pathway in mitochondria led to a decrease in cholesterol by 87.7% and in ergosterol by 55.1%, with a 669.6% increase in lanosterol (Figure 3), indicating that the post-squalene pathway beyond lanosterol was attenuated. Inhibition of the post-squalene pathway appeared to be regulated at a transcriptional level. For example, in the transcriptome, the transcripts encoding hSE and Erg7, which convert squalene into lanosterol, exhibited increased transcription in CEN-Cho-mMVA relative to CEN-Cho (Figure S6), which agreed with the increased level of lanosterol in CEN-Cho-mMVA. On the other hand, nearly all the transcripts (*Erg2*, *Erg3*, *Erg5*, *Erg6*, *Erg11*, *Erg25*, *Erg26*, *Erg27*, *StDWF5*, and *GgDHCR24*), except for *Erg24*, in the post-squalene pathway from lanosterol to either cholesterol or ergosterol were significantly down-regulated in CEN-Cho-mMVA, likely contributing to the decrease in production of cholesterol and ergosterol in CEN-Cho-mMVA (Figure S6). Moreover, in line with these transcriptome data, the proteome profile also revealed a decrease in protein abundance for those enzymes in the post-squalene pathway (Figure 4).

To understand how mitochondrial MVA pathway engineering down-regulated the post-squalene pathway, we extended the differentially gene expression analysis to glycolysis in cytosol and the TCA cycle in mitochondria. Generally, in view of the glycolysis process, especially at the stage from G3P to pyruvate, the proteome profile was not consistently correlated to the transcriptome (Figure S6 and Figure 4), indicative of post-transcriptional regulation occurring on *S. cerevisiae* glycolysis. A similar finding has also been reported by Daran et al. [27]. The 3′-UTRs of glycolytic genes harbor nutrient- and stress-responsive *cis*-acting elements, which regulate mRNA stability and thus uncouple mRNA and protein levels. In yeast, the 3′UTRs of *Pgk1* and *Pyk1* have been verified to modulate mRNA turnover through the TORC1/PKA signaling pathway under nutrient fluctuation [28]. In this study, the metabolic stress induced by mitochondrial MVA pathway engineering is likely to trigger such post-transcriptional regulation. Our proteomic analysis of CEN-Cho-mMVA showed strong inhibition of most of the enzymes in the downstream part of glycolysis (Figure 4), which would lead to decreased availability of pyruvate, which in turn would adversely affect the flux of pyruvate into the TCA cycle. Probably to cope with this down-regulation of glycolysis, the CEN-Cho-mMVA strain adapted to increase expression of Mpc1/2 (Figure 4), the enzyme complex that translocates pyruvate from cytosol to mitochondria, to maintain the TCA cycle function. Concomitantly, compared with the CEN-Cho strain, most of the enzymes in the TCA cycle of CEN-Cho-mMVA exhibited increased expression, revealed by both transcriptomic (Figure S6) and proteomic (Figure 4) analyses. The enhanced TCA cycle found in CEN-Cho-mMVA likely reflects a cellular response to the ectopic MVA pathway in mitochondria: to maintain metabolic homeostasis, the TCA cycle is up-regulated to compete with the relocated MVA pathway for the same mitochondrial acetyl-CoA pool. The decreased glycolysis and/or the increased TCA cycle would trigger increased oxidative phosphorylation in yeast [29,30]. Indeed, both *Idh2* and *Sdh3*, which are associated with oxidative phosphorylation [31,32], exhibited increased expression in CEN-Cho-mMVA (Figure S7). Given that mitochondrial oxidative phosphorylation is closely linked with the activation of several key nuclear sterol regulatory factors (Upc2, Hap1 and Mot3) [9,33,34], we compared the protein levels of Upc2, Hap1 and Mot3 between CEN-Cho and CEN-Cho-mMVA and found that Upc2 expression was almost completely suppressed in CEN-Cho-mMVA (Figure S8), which could lead to the down-regulation of the post-squalene pathway observed in this study.

## 4. Materials and Methods

### 4.1. Strains, Media, and Reagents

The background yeast strain used in this study is CEN.PK2-1C (*MATa*; *ura3*-52; *trp1*-289; *leu2*-3,112; *his3*Δ1; *MAL2*-8C; *SUC2*), obtained from the EUROSCARF (European *S. cerevisiae* Archive for Functional Analysis; accession No. 30000A) [35]. *Escherichia coli* DH5α (KT Life Technology Co., Ltd., Shenzhen, China) was used for plasmid construction and propagation. Recombinant *E. coli* strains were cultivated in Luria–Bertani (LB) medium supplemented with 100 μg/mL ampicillin or 50 μg/mL kanamycin.

Yeast cells were routinely grown in YPD medium consisting of 1% yeast extract, 2% peptone, and 2% glucose. Transformants harboring the gRNA-cas9 expression plasmid pCUT were selected on SD-Ura medium lacking uracil, while the engineered strains that had lost the pCUT plasmid were screened on SD-Ura medium containing 1 mg/mL 5-fluoroorotic acid (5-FOA). Leu- and Leu-Ura-deficient media were purchased from FunGenome Company (Beijing, China).

Squalene, nonadecanoic acid, cholesterol, ergosterol, and lanosterol standards (≥95% purity) were obtained from Yuanye Biotechnology Co., Ltd. (Shanghai, China). *n*-Hexane was supplied by CNW Technologies GmbH (Düsseldorf, Germany). Bis(trimethylsilyl)-trifluoroacetamide (BSTFA) was purchased from Regis Technologies, Inc. (Morton Grove, IL, USA). All other general reagents were obtained from Sinopharm Chemical Reagent Co., Ltd. (Shanghai, China) or Sangon Biotech Co., Ltd. (Shanghai, China).

### 4.2. Plasmid and Strain Construction

Seven yeast endogenous genes, including *tHmg1*, *Erg20*, *Acs1*, *Acs2*, *Erg10*, *pGal4-Gal4*, and *Erg6*, were amplified from the genomic DNA of CEN.PK 2-1C using PrimeSTAR MAX high-fidelity DNA polymerase (Takara, Japan). The genes which encode squalene synthase (hSQS) and squalene epoxidase (hSE) from *H. sapiens*, StDWF5 from *S. tuberosum*, and GgDHCR24 from *G. gallus* were codon-optimized and synthesized by Genewiz, Inc. (Suzhou, China). For expression in yeast mitochondria, a mitochondrial localization signal (MLS) sequence from the subunit IV of the yeast cytochrome oxidase [15] was cloned in fusion with the N-terminals of the targeted enzymes. Pairs of genes were cloned into the pESC-URA vector (Agilent Technologies, Santa Clara, CA, USA) and driven by the bidirectional GAL1-GAL10 promoter by using the ClonExpress II One Step Cloning Kit (Vazyme Biotech, Nanjing, China). The pCut plasmids containing the Cas9 and gRNA expression cassettes were prepared following the method previously published by Ro et al. [36]. Generally, the Cas9 expression cassette was amplified from the plasmid p414-TEF1p-Cas9-CYC1t (addgene number: 43802) and cloned into the *PuvII* site of pESC-URA, yielding an intermediate plasmid, pESC-URA-TEF1p-Cas9. The gRNA expression cassettes, harboring different 20 bp guide RNA sequences, were amplified from p426-SNR52p-gRNA.CAN1.Y-SUP4t (addgene number: 43803) via an overlapping extension PCR. The amplified gRNA expression cassettes were then cloned into the intermediate plasmid via the *KpnI* site to get the complete pCut constructs. All the primers used in this study are listed in Table S2, and all the plasmids made in this study are shown in Table S3.

For genomic integration, a mixture of 800 bp of 5′- and 3′-homology arms surrounding the targeted integration site, 0.5 µg of linear donor DNA containing the expression cassettes of the targeted genes, and 0.5 µg of the pCut plasmid were co-transformed into yeast cells through the lithium acetate transformation protocol [37]. The transformed yeast cells were grown on SD-Ura plates, and correct integration was verified by PCR analysis of genomic DNA. Positive colonies were then streaked on SC–5-FOA plates to remove the pCUT plasmid before the next round of integration. The yeast strains constructed in this study are listed in Table S4.

### 4.3. Shake Flask Fermentation of Engineered Yeast

Ten colonies per yeast strain were inoculated into 5 mL of YPD media with 2% glucose as a carbon source and cultured overnight at 30 °C with shaking at 220 rpm. The seed culture was then transferred into a 100 mL shake flask containing 20 mL of YPD medium and cultivated under the same conditions until the optical density at 600 nm (OD_600_) reached ~0.8. Cells were harvested by centrifugation, the supernatant was discarded, and the pellet was washed three times with sterile water. The washed cells were re-suspended in 20 mL of 2% Gal-YPD medium and induced at 30 °C for 5 days.

### 4.4. Sterol Extraction and Quantification

Approximately 0.1 g of fresh yeast cells was re-suspended in 3 mL of 25% KOH in methanol, with 20 µg of nonadecanoic acid (1 mg/mL in methanol) added as an internal standard. The mixture was sonicated in a water bath for 2.0 h to disrupt the cells, followed by saponification at 80 °C for 1 h. Sterols were extracted three times with 2 mL of n-hexane. The combined organic phases were washed once with 3 mL of ultrapure water and dried under vacuum. The dried residue was derivatized by adding 50 µL of N,O-Bis (trimethylsilyl) trifluoroacetamide (BSTFA) and incubated at 80 °C for 1 h. Subsequently, 100 µL of chloroform was added and vortexed, and the supernatant was used for GC–MS analysis.

GC–MS analysis was performed on a Shimadzu QP-2010 Plus system (Shimadzu, Kyoto, Japan) equipped with a DB-5MS capillary column (30 m × 0.25 mm × 0.25 µm). Nitrogen was used as a carrier gas at a flow rate of 1.5 mL/min, with a 1 µL injection volume. The injector and detector temperatures were set to 250 °C. The oven temperature program was as follows: the initial temperature of 80 °C was held for 2 min; it was then ramped at 30 °C/min to 310 °C; finally it was held at 300 °C for 15 min. Mass spectra were recorded in scan mode over mass range *m*/*z* 50–600.

### 4.5. Comparative Transcriptomic and Proteomic Analyses

Transcriptomic and proteomic analyses were performed on the engineered yeast strains to examine the changes in protein and mRNA abundance due to the cytosolic and mitochondrial engineering of the MVA pathway.

For transcriptomic analysis, total RNA was extracted from 20 mg of yeast cells using TRIzol^®^ Reagent (Thermo Fisher Scientific, Waltham, MA, USA), and RNA quality was assessed by agarose gel electrophoresis and NanoDrop/Qubit measurements. mRNA was enriched using poly-T magnetic beads, and sequencing libraries were prepared with the TruSeq™ RNA Sample Preparation Kit (Illumina, San Diego, CA, USA). Libraries were sequenced on the HiSeq 4000 platform (Illumina, CA, USA). Data processing, including normalization, principal component analysis (PCA), clustering, and analysis of differentially expressed genes, was performed using Cell Ranger v7.1.0 and the Seurat package (Wilcoxon Rank-Sum test) [38]. Gene annotation was conducted with SingleR [39].

For proteomic analysis, yeast cells were lysed with protein lysis buffer containing 8 M urea, 1% SDS, and a protease inhibitor cocktail, and total protein was precipitated with 100 mM triethylammonium bicarbonate buffer (TEAB), reduced with 10 mM Tris(2-carboxyethyl) phosphine (TCEP) at 37 °C for 60 min, and alkylated with 40 mM iodoacetamide (IAM) at room temperature in the dark for 40 min, followed by trypsin digestion. Peptides were desalted using HLBC columns (HLB C18, Waters, Milford, MA, USA), reconstituted, and analyzed via data-independent acquisition (DIA) mass spectrometry on a Vanquish Neo LC coupled with Orbitrap Astral MS (Thermo Fisher Scientific, Waltham, MA, USA). Data searching was performed using Spectronaut™ 19 (Biognosys AG, Schlieren, Switzerland), and protein quantification was conducted using the MaxLFQ algorithm [40]. Differentially expressed proteins (DEPs) were defined as those with *p* < 0.05 and fold change > 1.2. Functional annotation included Gene Ontology (GO) enrichment, Kyoto Encyclopedia of Genes and Genomes (KEGG) pathway mapping, and protein–protein interaction network construction using STRING v11.5 [41].

## 5. Conclusions

The data of this study suggest that the mitochondrial MVA pathway engineering strategy is not appropriate for improving production of post-squalene chemicals, at least for steroidal compounds, although it has been successfully applied to achieve high squalene production in yeast [16]. Decreased glycolysis and increased TCA cycle in response to mitochondrial MVA targeting, resulting in oxidative phosphorylation stress, may form a challenge in applying this strategy to boost the post-squalene pathway in yeast.

## Data Availability

The RNA sequencing data of CEN-Cho and CEN-Cho-mMVA have been deposited in the NCBI SRA database under accession number PRJNA1420251. The mass spectrometry proteomic data of CEN-Cho and CEN-Cho-mMVA have been deposited in the PRIDE database with accession number PXD074200.

## Supplementary Materials

The following supporting information can be downloaded at: https://www.mdpi.com/article/10.3390/molecules31121999/s1. Table S1: Mapping statistics of clean transcriptomic reads against the reference genome; Table S2: The primers used in this study; Table S3: The plasmids prepared in this study; Table S4: The yeast strains constructed by this study; Figure S1: Comparison of the growth curves of CEN-Cho and CEN-Cho-mMVA; Figure S2: Principal component analysis (PCA) of transcriptomic samples from CEN-Cho (TreatA) and CEN-Cho-mMVA (TreatB); Figure S3: Principal component analysis (PCA) of proteomic samples from CEN-Cho (TreatA) and CEN-Cho-mMVA (TreatB); Figure S4: The KEGG pathway enrichment analysis of the differentially expressed genes between CEN-Cho (Treatment A) and CEN-Cho-mMVA (Treatment B); Figure S5: KEGG pathway enrichment analysis of differentially expressed proteins from cells harvested at 120 h for CEN-Cho (Treatment A) and CEN-Cho-mMVA (Treatment B); Figure S6: Comparison of CEN-Cho and CEN-Cho-mMVA in the expression of transcripts related to the processes of glycolysis, TCA cycle, and cytosolic acetyl-CoA supply, and the biosynthetic steps up to squalene, cholesterol and ergosterol; Figure S7: Comparison of the abundance of Idh2, Lsc2, Sdh1, Sdh2, Sdh3 and Sdh4 expressed in the 120 h harvested cells of CEN-Cho and CEN-Cho-mMVA; Figure S8: Comparison of the abundance of Upc2, Hap1 and Mot3 expressed in the 120 h harvested cells of CEN-Cho and CEN-Cho-mMVA.

## Abbreviations

The following abbreviations are used in this manuscript:

Erg7	Lanosterol synthase
Erg11	Lanosterol 14α-demethylase
Erg24	Sterol C-14 reductase
Erg25	C-4 methylsterol oxidase
Erg26	C-3 sterol dehydrogenase/C-4 decarboxylase
Erg27	3-Ketosteroid reductase
Erg2	C-8 sterol isomerase
Erg3	C-5 sterol desaturase
Erg6	S-Adenosylmethionine-dependent C-24 sterol methyltransferase
Erg4	Sterol C-24(28) reductase
Erg5	C-22 sterol desaturase
StDWF5	Δ7-Sterol reductase
GgDHCR24	Δ24-Dehydrocholesterol reductase.
