# Supplementary material for: Mitochondrial Targeting of MVA Pathway Triggers Severe Inhibition of Post-Squalene Cholesterol Biosynthesis: Transcriptomic and Proteomic Insights in Yeast
Source: Molecules. 2026 Jun 7;31(12):1999. doi: 10.3390/molecules31121999 (PMC13304375; doi:10.3390/molecules31121999)
Supplement: Supplementary file 1 [file molecules-31-01999-s001.zip › molecules-4323426-supplementary.pdf]

Supplementary data for

Mitochondrial Targeting of MVA Pathway Triggers Severe Inhibition of Post-Squalene Cholesterol Biosynthesis: Transcriptomic and Proteomic Insights in Yeast

Nan Tang, Yuliang Xu, Changfu Li and Yansheng Zhang*

Shanghai Key Laboratory of Bio-Energy Crops, Synthetic Biology Research Center, School of Life Sciences, Shanghai University, Shanghai 200444, China. nantang@shu.edu.cn (N.T.); xyltta0ssaw@shu.edu.cn (Y.X.); changfuli@shu.edu.cn (C.L.).

* Correspondence: zhangys1@shu.edu.cn (Y.Z.)

Table S1. Mapping statistics of clean transcriptomic reads against the reference genome.

Sample	Raw Reads	Clean Reads	Total Mapped	Multipple Mapped	Uniquely Mapped
CEN-Cho_1	44066500	44066500	36420668 (83.15%)	2690746 (6.14%)	33729922 (77.01%)
CEN-Cho_2	43966728	43966728	36281648 (83.06%)	2641778 (6.05%)	33639870 (77.01%)
CEN-Cho_3	42243640	42243640	34566638 (82.42%)	2428370 (5.79%)	32138268 (76.63%)
CEN-Cho-mMVA_1	39714282	39714282	37512155 (95.14%)	4439973 (11.26%)	33072182 (83.88%)
CEN-Cho-mMVA_2	39610322	39610322	37234724 (94.63%)	4292576 (10.91%)	32942148 (83.72%)
CEN-Cho-mMVA_3	43508882	43508882	41288111 (95.38%)	5224132 (12.07%)	36063979 (83.31%)

Table S2. The primers used in this study.

Nos.	Nucleotide sequence (5′ to 3′)	Used for
1	AAAAGTAAGAATTTTTGAAAATTCGAATTCA TGGCTGCAGACCAATTGG	preparing the plasmid ‘pESC-URA-tHMG1-hSE’
2	CCAAACCTCTGGCGAAGAATTGTTAATTAATT AGGATTTAATGC	
3	CTTTAACGTCAAGGAGAAAAAACCCCGGATC CATGGCTACTGCTGCTTGTACTTC	
4	CTTCGGAAATCAACTTCTGTTCCATGTCGACT CAATGAACCATATACTTCATCTC	
5	CTTTAACGTCAAGGAGAAAAAACCCCGGATC CATGGCTTCAGAAAAAGAAATTAG	preparing the plasmid ‘pESC-URA-tHMG1-ERG20’
6	CTTCGGAAATCAACTTCTGTTCCATGTCGACC TATTTGCTTCTCTTGTAAAC	
7	AAAAGTAAGAATTTTTGAAAATTCGAATTCA TGTCTCAGAACGTTTACATTG	preparing the plasmid ‘pESC-URA-ERG10-ACS1’
8	CCAAACCTCTGGCGAAGAATTGTTAATTAAT CATATCTTTTCAATGACAATA	
9	CTTTAACGTCAAGGAGAAAAAACCCCGGATC CATGTCGCCCTCTGCCGTACAATC	
10	CTTCGGAAATCAACTTCTGTTCCATGTCGACT TACAACTTGACCGAATCAATTAG	
11	AAAAGTAAGAATTTTTGAAAATTCGAATTCA	preparing the plasmid

Nos.	Nucleotide sequence (5′ to 3′)	Used for
	TGACAATCAAGGAACATAAAG	'pESC-URA-hSQS-ACS2'
12	CCAAACCTCTGGCGAAGAATTGTTAATTAATTATTTCTTTTTTTGAGAG	
13	CTTTAACGTCAAGGAGAAAAAACCCCGGATCCATGGACCAAGACTCTTTGTCCTC	
14	CTTCGGAAATCAACTTCTGTTCCATGTCGACTCAGTTCTGGGTTCTAATGG	
15	GCGAAGAATTGTTAATTAAGAGCTCAGATCTGACAGCATTCGCCCAGTA	preparing the plasmid 'pESC-URA-pGAL4-GAL4'
16	CGGATCTTAGCTAGCCGCGGTACCTTACTCTTTTTTTGGGTTTGG	
17	AAAAGTAAGAATTTTTGAAAATTCGAATTCATGGCCGAATCTCAATTGG	preparing the plasmid 'pESC-URA-StDWF5-GgDHCR24'
18	CCAAACCTCTGGCGAAGAATTGTTAATTAATCAGTAGATGCCTGGAATAAC	
19	TACTTTAACGTCAAGGAGAAAAAACCCCGGATCCATGTCAGCTGTTTGGTCTTTAGGTG	
20	CTTCTTCGGAAATCAACTTCTGTTCCATGTCGACTTAATGTCTTGCAGCCTTAC	
21	AAAAGTAAGAATTTTTGAAAATTCGAATTCATGCAATCTACTACAAGCGTAAAG	preparing the plasmid 'pESC-Leu2d-AaCPR-Re-ERG6'
22	CTTGACCAAACCTCTGGCGAAGAATTGTTAATTAACTACCAAACATCTCTCAAATACC	
23	CTTCGGAAATCAACTTCTGTTCCATGTCGACATGAGTGAAACAGAATTGAGAAAAAG	
24	CTTTAACGTCAAGGAGAAAAAACCCCGGATCCTTATTGAGTTGCTTCTTGGG	
25	GCTTTAATTTGCGGCCGGTACTCTTTGAAAAGATAATGTATGATTATGCTTTC	preparing the plasmid 'pCUT-gre3'
26	AACCGGGCTTGATTCTACAACCAGATCATTTATCTTTCACTGC	
27	GATCTGGTTGTAGAATCAAGCCCGGTTTTAGAGCTAGAAATAGC	
28	TCGCTATTACGCCAGCCTAGGAGACATAAAAAACAAAAAAAGCACCAC	
29	GCTTTAATTTGCGGCCGGTACTCTTTGAAAAGATAATGTATGATTATGCTTTC	preparing the plasmid 'pCUT-adh3'
30	AACGTCACCAGCTTTCAAGTCTGGATCATTTATCTTTCACTGC	
31	GATCCAGACTTGAAAGCTGGTGACGTTTTAGAGCTAGAAATAGC	
32	TCGCTATTACGCCAGCCTAGGAGACATAAAA	

Nos.	Nucleotide sequence (5′ to 3′)	Used for
	AACAAAAAAAGCACCAC	
33	GCTTTAATTTGCGGCCGGTACTCTTTGAAAAGATAATGTATGATTATGCTTTC	preparing the plasmid 'pCUT-adh4'
34	AACGCTTTGACTGCTGCTACTGGGATCATTTATCTTTCACTGC	
35	GATCCCAGTAGCAGCAGTCAAAGCGTTTTAGAGCTAGAAATAGC	
36	TCGCTATTACGCCAGCCTAGGAGACATAAAAAACAAAAAAAGCACCAC	
37	GCTTTAATTTGCGGCCGGTACTCTTTGAAAAGATAATGTATGATTATGCTTTC	preparing the plasmid 'pCUT-gpd1'
38	AACAAGTCGCTCAAGAACACTGGGATCATTTATCTTTCACTGC	
39	GATCCCAGTGTTCTTGAGCGACTTGTTTTAGAGCTAGAAATAGC	
40	TCGCTATTACGCCAGCCTAGGAGACATAAAAAACAAAAAAAGCACCAC	
41	GCTTTAATTTGCGGCCGGTACTCTTTGAAAAGATAATGTATGATTATGCTTTC	preparing the plasmid 'pCUT-gal1-7'
42	AACGCCGATCGTGGCGCCTATATGATCATTTATCTTTCACTGC	
43	GATCATATAGGCGCCACGATCGGCGTTTTAGAGCTAGAAATAGC	
44	TCGCTATTACGCCAGCCTAGGAGACATAAAAAACAAAAAAAGCACCAC	
45	GCTTTAATTTGCGGCCGGTACTCTTTGAAAAGATAATGTATGATTATGCTTTC	preparing the plasmid 'pCUT-delta15'
46	AACATGCTCTGTTGTTCGGATTTGATCATTTATCTTTCACTGC	
47	GATCAAATCCGAACAACAGAGCATGTTTTAGAGCTAGAAATAGC	
48	TCGCTATTACGCCAGCCTAGGAGACATAAAAAACAAAAAAAGCACCAC	
49	GCTTTAATTTGCGGCCGGTACTCTTTGAAAAGATAATGTATGATTATGCTTTC	preparing the plasmid 'pCUT-ARS1014'
50	AACCCTAGTATCCCGTGAATATGGATCATTTATCTTTCACTGC	
51	GATCCATATTCACGGGATACTAGGGTTTTAGAGCTAGAAATAGC	
52	TCGCTATTACGCCAGCCTAGGAGACATAAAAAACAAAAAAAGCACCAC	
53	GCTTTAATTTGCGGCCGGTACTCTTTGAAAAG	preparing the plasmid

Nos.	Nucleotide sequence (5′ to 3′)	Used for
	ATAATGTATGATTATGCTTTC	‘pCUT-ARS911’
54	AACGGGAAACAAGACAATATTACGATCATTTATCTTTCACTGC	
55	GATCGTAATATTGTCTTGTTTCCCGTTTTAGAGCTAGAAATAGC	
56	TCGCTATTACGCCAGCCTAGGAGACATAAAAAACAAAAAAAGCACCAC	
57	GCTTTAATTTGCGGCCGGTACTCTTTGAAAAGATAATGTATGATTATGCTTTC	preparing the plasmid ‘pCUT-ARS1406’
58	AACTGCATTCCCATCAGAACCGTGATCATTTATCTTTCACTGC	
59	GATCACGGTTCTGATGGGAATGCAGTTTTAGAGCTAGAAATAGC	
60	TCGCTATTACGCCAGCCTAGGAGACATAAAAAACAAAAAAAGCACCAC	
61	GCTTTAATTTGCGGCCGGTACTCTTTGAAAAGATAATGTATGATTATGCTTTC	preparing the plasmid ‘pCUT-ARS1531’
62	AACCCCTGAAAGTTGCCGAGGCCGATCATTTATCTTTCACTGC	
63	GATCGGCCTCGGCAACTTTCAGGGGTTTTAGAGCTAGAAATAGC	
64	TCGCTATTACGCCAGCCTAGGAGACATAAAAAACAAAAAAAGCACCAC	
65	CGCTGTCTTGATTCTTGTGAGC	integrating the expression cassette onto the *gre3* locus on Chr VIII
66	GCATGAGGTCGCTCCAATTCAGACTTTTGCCTGATCCAGCCAG	
67	CTGGCTGGATCAGGCAAAAGTCTGAATTGGAGCGACCTCATGC	
68	AGATGTCTTCACTGGTTACTCTTCTTCGAGCGTCCCAAAACCT	
69	AGGTTTTGGGACGCTCGAAGAAGAGTAACCAGTGAAGACATCT	
70	CCTGGTGGAACATCCTAGAACG	
71	TTGTGACACGTCAGTGCAGG	integrating the expression cassette onto the *adh3* locus on Chr XIII
72	GCATGAGGTCGCTCCAATTCAGTGTTGACGTTCTCAACATGATGG	
73	CCATCATGTTGAGAACGTCAACACTGAATTGGAGCGACCTCATGC	
74	GGGTGCGTAACACGCTATTACTTCGAGCGTCCCAAAACCTTC	
75	GAAGGTTTTGGGACGCTCGAAGTAATAGCGTGTTACGCACCC	

Nos.	Nucleotide sequence (5′ to 3′)	Used for
76	CCAATGCCTGTAACCCTTCG	
77	ACATCCTTGTCGAGCCTTGG	integrating the expression cassette onto the *gpd1* locus on Chr IV
78	GCATGAGGTCGCTCCAATTCAGATGTGGCTCTGTCGAAGACTTCC	
79	GGAAGTCTTCGACAGAGCCACATCTGAATTGGAGCGACCTCATGC	
80	ATGTCTGCTGCTGCTGATAGACTTCGAGCGTCCCAAAACCTTC	
81	GAAGGTTTTGGGACGCTCGAAGTCTATCAGCAGCAGCAGACAT	
82	GTCCTCGGTAGATCAGGTCAGT	
83	GGATCCCCGGAGGCCTTCAA	integrating the expression cassette onto the *adh4* locus on Chr VII
84	GCATGAGGTCGCTCCAATTCAGCCGGAGAGACCAATAGCTGC	
85	GCAGCTATTGGTCTCTCCGGCTGAATTGGAGCGACCTCATGC	
86	GCAGGCATCATGCATGGCGTCTTCGAGCGTCCCAAAACCT	
87	AGGTTTTGGGACGCTCGAAGACGCCATGCATGATGCCTGC	
88	GCGCATGTGAATGACACACG	
89	GTGAAGCAATTAGACGCAAACG	integrating the expression cassette onto the *ARS1014* locus on Chr X
90	GCATGAGGTCGCTCCAATTCAGACATACTACGAGTTCTCCTCGAG	
91	CTCGAGGAGAACTCGTAGTATGTCTGAATTGGAGCGACCTCATGC	
92	GAAATTTGTAACATGCTGACCTCTTCGAGCGTCCCAAAACCT	
93	AGGTTTTGGGACGCTCGAAGAGGTCAGCATGTTACAAATTTC	
94	GGCATGCAATGTATATGGCT	
95	GACTGCCTCTTGATGTTATGCCA	integrating the expression cassette onto the ARS1531 locus on Chr XV
96	GCATGAGGTCGCTCCAATTCAGGAAAGTTGCCGAGGCCAAATG	
97	CATTTGGCCTCGGCAACTTTCCTGAATTGGAGCGACCTCATGC	
98	GGTATATACGTTCAAGCCCCCCTTCGAGCGTCCCAAAACCT	
99	AGGTTTTGGGACGCTCGAAGGGGGGCTTGAACGTATATACC	
100	GGATGGCAGAACCGATACTAATG	
101	TCCTCGCGCTTGTCTACTA	integrating the expression

Nos.	Nucleotide sequence (5′ to 3′)	Used for
102	ATGAGGTCGCTCCAATTCAGGCATTGGGCAG CTGTCTATATG	cassette onto the *gal1-7* locus on Chr II
103	CATATAGACAGCTGCCCAATGCCTGAATTGG AGCGACCTCAT	
104	AGATAATGAATCTGACCATCCTTCGAGCGTC CCAAAACCT	
105	AGGTTTTGGGACGCTCGAAGGATGGTCAGAT TCATTATCT	
106	GAGGGTAATAACATAGGTGCAG	
107	GGAAGCTGAAATGCAAAGATCG	integrating the expression cassette onto the *delta15* locus on Chr XVI
108	GCATGAGGTCGCTCCAATTCAGCGCAAACAA ACTTAAATATATGC	
109	GCATATATTTAAGTTTGTTTGCGCTGAATTGG AGCGACCTCATGC	
110	GAAAGAAAAACTAACACATTAATGTAGCTTC GAGCGTCCCAAAACCTTC	
111	GAAGGTTTTGGGACGCTCGAAGCTACATTAA TGTGTTAGTTTTTCTTTC	
112	CCGATAACGCCAGGCGCCTT	
113	TAGTGGAGGCAAGGTTGCAT	integrating the expression cassette onto the *ARS911* locus on Chr IX
114	GCATGAGGTCGCTCCAATTCAGTTATGCCCAT TCAACATCCG	
115	CGGATGTTGAATGGGCATAACTGAATTGGAG CGACCTCATGC	
116	TTTTCATTTACTTCTCCAGGGCTTCGAGCGTCC CAAAACCT	
117	AGGTTTTGGGACGCTCGAAGCCCTGGAGAAG TAAATGAAAA	
118	CGGAAACATTATACTCAAGTCGC	
119	GTTGGTATTCTCGATAGGCAGC	integrating the expression cassette onto the *ARS1406* locus on Chr IX
120	GCATGAGGTCGCTCCAATTCAGCCCATCAGA ACCGTAAACCTTG	
121	CAAGGTTTACGGTTCTGATGGGCTGAATTGGA GCGACCTCATGC	
122	CAAGACTAGATTCCCCCCTGCTTCGAGCGTCC CAAAACCT	
123	AGGTTTTGGGACGCTCGAAGCAGGGGGGAAT CTAGTCTTG	
124	GCGTCCTTATCGAAAGGAAC	
125	CCTCCAGCGAATCTACACTTACAGC	integrating the expression cassette onto the *ATF2* locus
126	GCATGAGGTCGCTCCAATTCAGGACTGCGAG AAGATTAAATCC	

Nos.	Nucleotide sequence (5′ to 3′)	Used for
127	GGATTTAATCTTCTCGCAGTCCTGAATTGGAG CGACCTCATGC	on Chr VII
128	CCGAACGCAAATCTCAAGGCGCTTCGAGCGT CCCAAAACCTTC	
129	GAAGGTTTTGGGACGCTCGAAGCGCCTTGAG ATTTGCGTTCGG	
130	CAATTCAGGTGCAACGTTAACAGC	

Table S3. The plasmids prepared in this study.

Plasmid name	Description	Resource
pESC-URA	Yeast expression vector; Amp	Cat. No. 217454, Agilent Technologies/Stratagene
pCUT	The plasmid containing the expression cassettes of Cas9 and guide RNA, which was constructed based on the pESC-URA backbone	[1]
pESC-URA-tHmg1-hSE	The pESC-URA bearing *tHmg1* and *hSE*	This study
pESC-URA-tHmg1-Erg20	The pESC-URA bearing *tHmg1* and *Erg20*	This study
pESC-URA-Erg10-Acs1	The pESC-URA bearing *Erg10* and *Acs1*	This study
pESC-URA-hSQS-Acs2	The pESC-URA bearing hSQS and Acs2	This study
pESC-URA-pGAL4-GAL4	The pESC-URA bearing *pGAL4-GAL4*	This study
pESC-URA-StDWF5-GgDHCR24	The pESC-URA bearing *StDWF5* and *GgDHCR24*	This study
pESC-URA-MLS-tHmg1-Erg10	The pESC-URA bearing *MLS-tHmg1* and *MLS-Erg10*	Synthesized by the GENEWIZ Inc.
pESC-URA-MLS-tHmg1-Erg12	The pESC-URA bearing *MLS-tHmg1* and *MLS-Erg12*	Synthesized by the GENEWIZ Inc.

Plasmid name	Description	Resource
pESC-URA-MLS-Erg19-Erg13	The pESC-URA bearing *MLS-Erg19* and *MLS-Erg13*	Synthesized by the GENEWIZ Inc.
pESC-URA-MLS-Erg19-Erg8	The pESC-URA bearing *MLS-Erg19* and *MLS-Erg8*	Synthesized by the GENEWIZ Inc.
pESC-Leu2d-AaCPR-*Re*-Erg6	The pESC-Leu2d bearing *AaCPR* and *Re-Erg6*	Synthesized by the GENEWIZ Inc.
pCUT-gre3	The pCUT targeting the *gre3* locus on Chr VIII	This study
pCUT-adh3	The pCUT targeting the *adh3* locus on Chr XIII	This study
pCUT-adh4	The pCUT targeting the *adh4* locus on Chr VII	This study
pCUT-gpd1	The pCUT targeting the *gpd1* locus on Chr IV	This study
pCUT-gal1-7	The pCUT targeting the *gal1-7* locus on Chr II	This study
pCUT-delta15	The pCUT targeting the *delta15* locus on Chr XVI	This study
pCUT-ARS1014	The pCUT targeting the *ARS1014* locus on Chr X	This study
pCUT-ARS911	The pCUT targeting the *ARS911* locus on Chr IX	This study
pCUT-ARS1406	The pCUT targeting the *ARS1406* locus on Chr IX	This study
pCUT-ARS1531	The pCUT targeting the *ARS1531* locus on Chr XV	This study
pCUT-ATF2	The pCUT targeting the *ATF2* locus on Chr VII	This study

Table S4. The yeast strains constructed by this study.

Strains	Host strains	Descriptions	Resources
CEN.PK2-1C		*MATa*; *ura3*-52; *trp1*-289; *leu2*-3,112; *his3*Δ1;<br>*MAL2*-8C; *SUC2*	[2]
CEN-Cho	CEN.PK2-1C	*MATa*; *ura3*-52; *trp1*-289; *leu2*-3,112; *his3*Δ1;<br>*MAL2*-8C; *SUC2*<br>*ARS1406::pGAL1-hSQS-pGAL10-Acs2;*<br>*ARS1531::pGAL1-StDWF5-pGAL10-*<br>*GgDHCR24;*<br>*ARS911::pGAL1-Acs1-pGAL10-Erg10；*<br>*Delta15::pGAL1-tHmg1-pGAL10-Erg20*<br>*ARS1014::pGAL1-hSE-pGAL10-tHmg1*<br>*ATF2::pGAL1-AaCPR-pGAL10-Re-Erg6;*<br>*Gal1-7:: pGAL1- PGAL4-GAL4*	This study
CEN-Cho-<br>mMVA	CEN-Cho	*MATa*; *ura3*-52; *trp1*-289; *leu2*-3,112; *his3*Δ1;<br>*MAL2*-8C; *SUC2*<br>*ARS1406::pGAL1-hSQS-pGAL10-Acs2;*<br>*ARS1531::pGAL1-StDWF5-pGAL10-*<br>*GgDHCR24;*<br>*ARS911::pGAL1-Acs1-pGAL10-Erg10；*<br>*Delta15::pGAL1-tHmg1-pGAL10-Erg20*<br>*ARS1014::pGAL1-hSE-pGAL10-tHmg1;*<br>*adh4::pGAL1-**MLS**-Erg10-pGAL10-**MLS**-*<br>*tHmg1;*<br>*adh3::pGAL1-**MLS**-Erg13-pGAL10-**MLS**-Erg19;*<br>*gpd1::pGAL1-**MLS**-Erg12-pGAL10-**MLS**-tHmg1*<br>*gre3::pGAL1-**MLS**-Erg8-pGAL10-**MLS**-Erg19*<br>*ATF2::pGAL1-AaCPR-pGAL10-Re-Erg6;*<br>*Gal1-7:: pGAL1-*P_{GAL4}*-GAL4*	This study

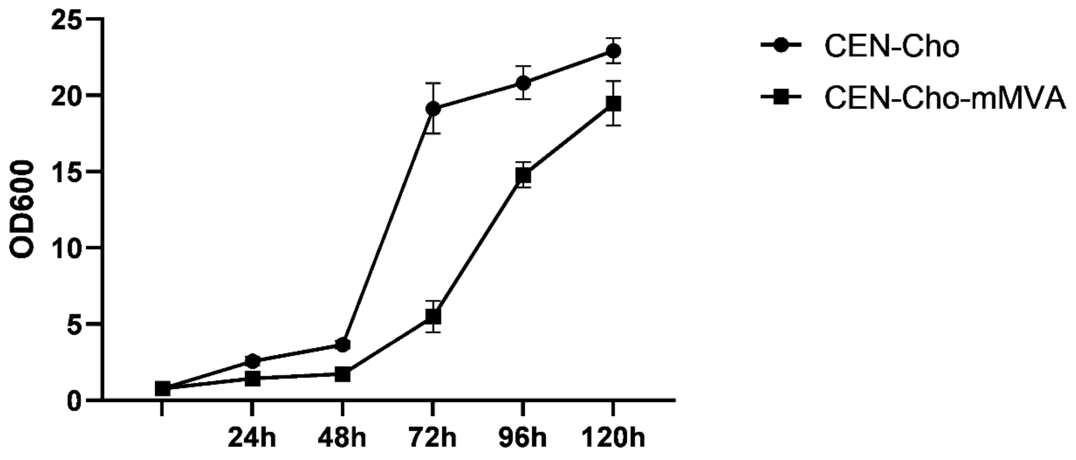

Figure S1. Comparison of the growth curves of CEN-Cho and CEN-Cho-mMVA. For each strain, a mixture of ten colonies with a similar size were picked up from a YPD plate, grown in a YPD liquid medium for 48 h, and then was cultivated in an induction medium containing 2% galactose for another three days. Growth was monitored every 24 hours by measuring optical density at 600 nm.

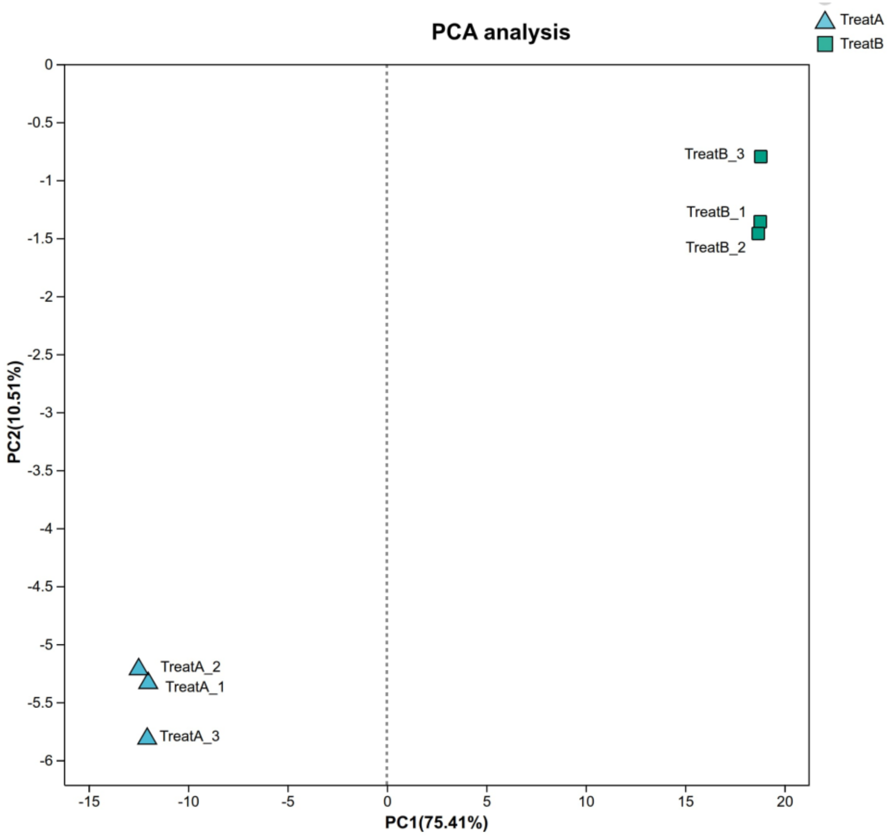

Figure S2. Principal component analysis (PCA) of transcriptomic samples from CEN-Cho (TreatA) and CEN-Cho-mMVA (TreatB). Dimensionality reduction was performed to visualize sample variation in the PCA plot. The distance between data points reflects sample similarity: shorter distances indicate higher similarity.

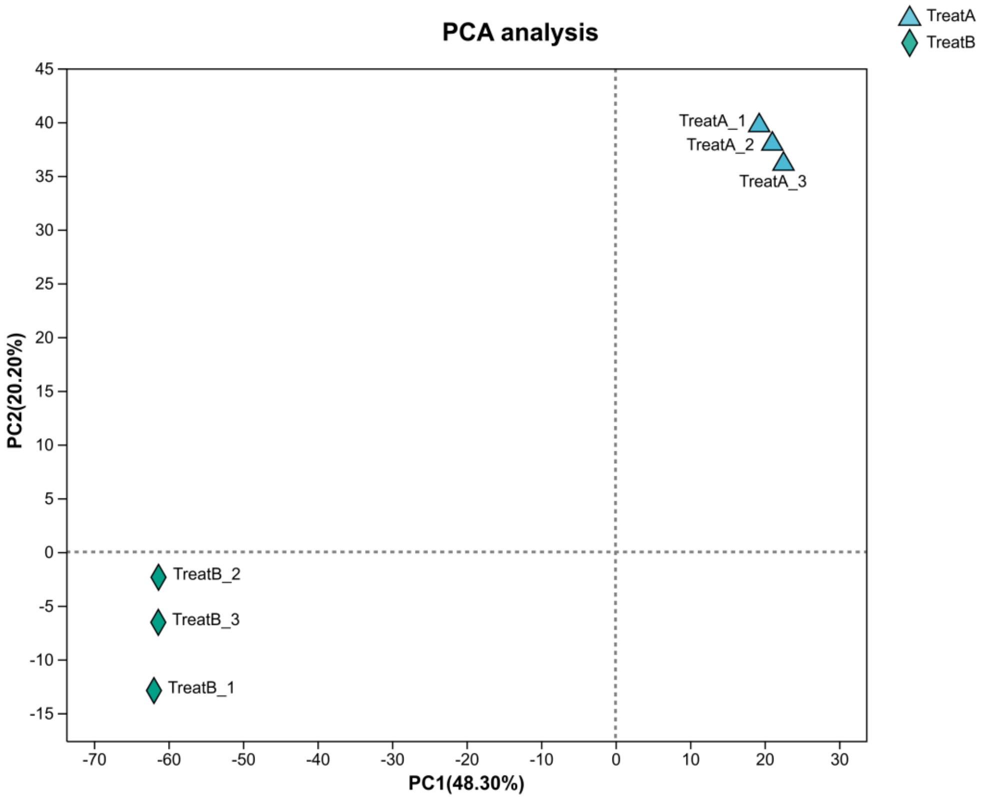

Figure S3. Principal component analysis (PCA) of proteomic samples from CEN-Cho (TreatA) and CEN-Cho-mMVA (TreatB). Dimensionality reduction was performed to visualize sample variation in the PCA plot. The distance between data points reflects sample similarity: shorter distances indicate higher similarity.

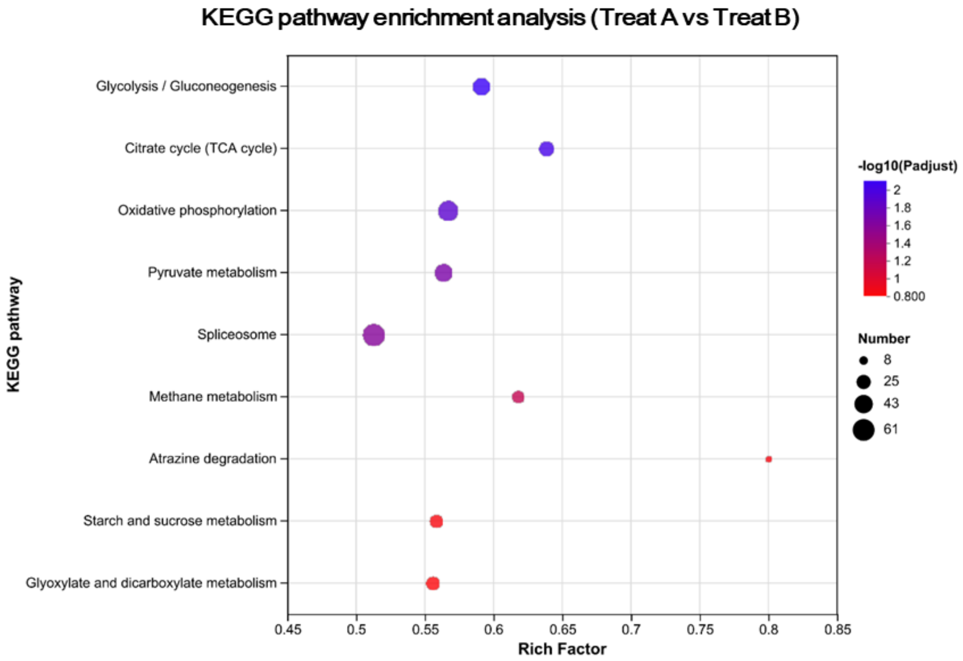

Figure S4. The KEGG pathway enrichment analysis of the differentially expressed genes between CEN-Cho (Treatment A) and CEN-Cho-mMVA (Treatment B). The yeast cells harvested at the 120 h time point of Figure S1 were subjected to transcriptome analysis. The analysis was performed in triplicate. For each KEGG pathway, the Rich Factor was calculated as the ratio of the number of differentially expressed genes annotated to the pathway to the total number of genes in the pathway, serving as an indicator of the proportion of pathway genes affected by the treatment.

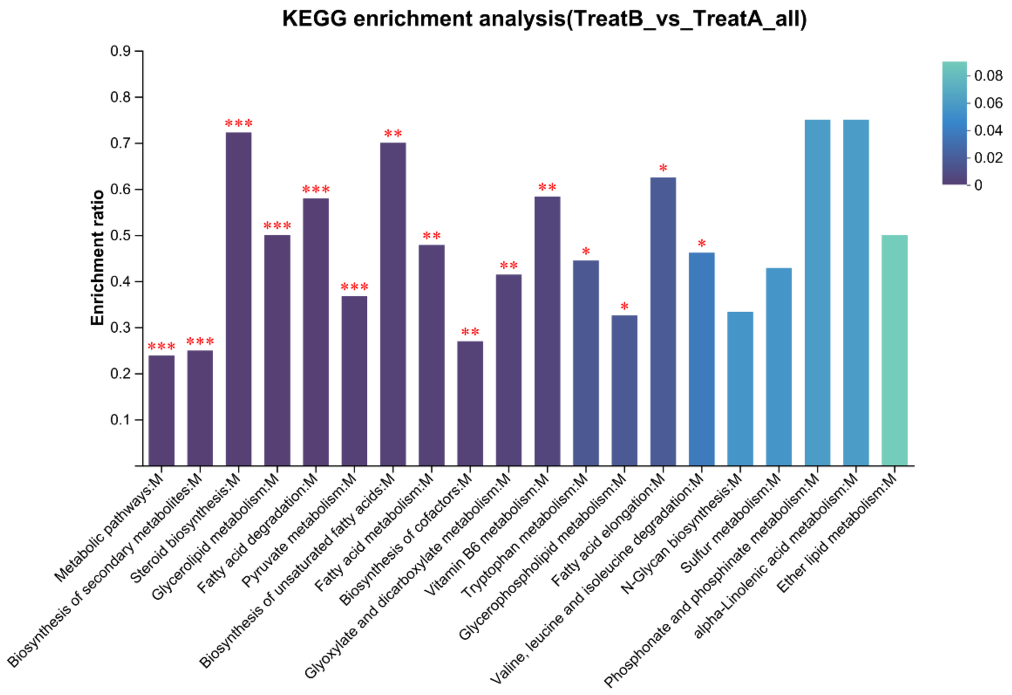

Figure S5. KEGG pathway enrichment analysis of differentially expressed proteins from cells harvested at 120 h for CEN-Cho (Treatment A) and CEN-Cho-mMVA (Treatment B). The vertical axis shows the enrichment ratio, defined as the number of proteins enriched in a pathway divided by the total number of proteins annotated to that pathway. A higher ratio represents stronger enrichment.

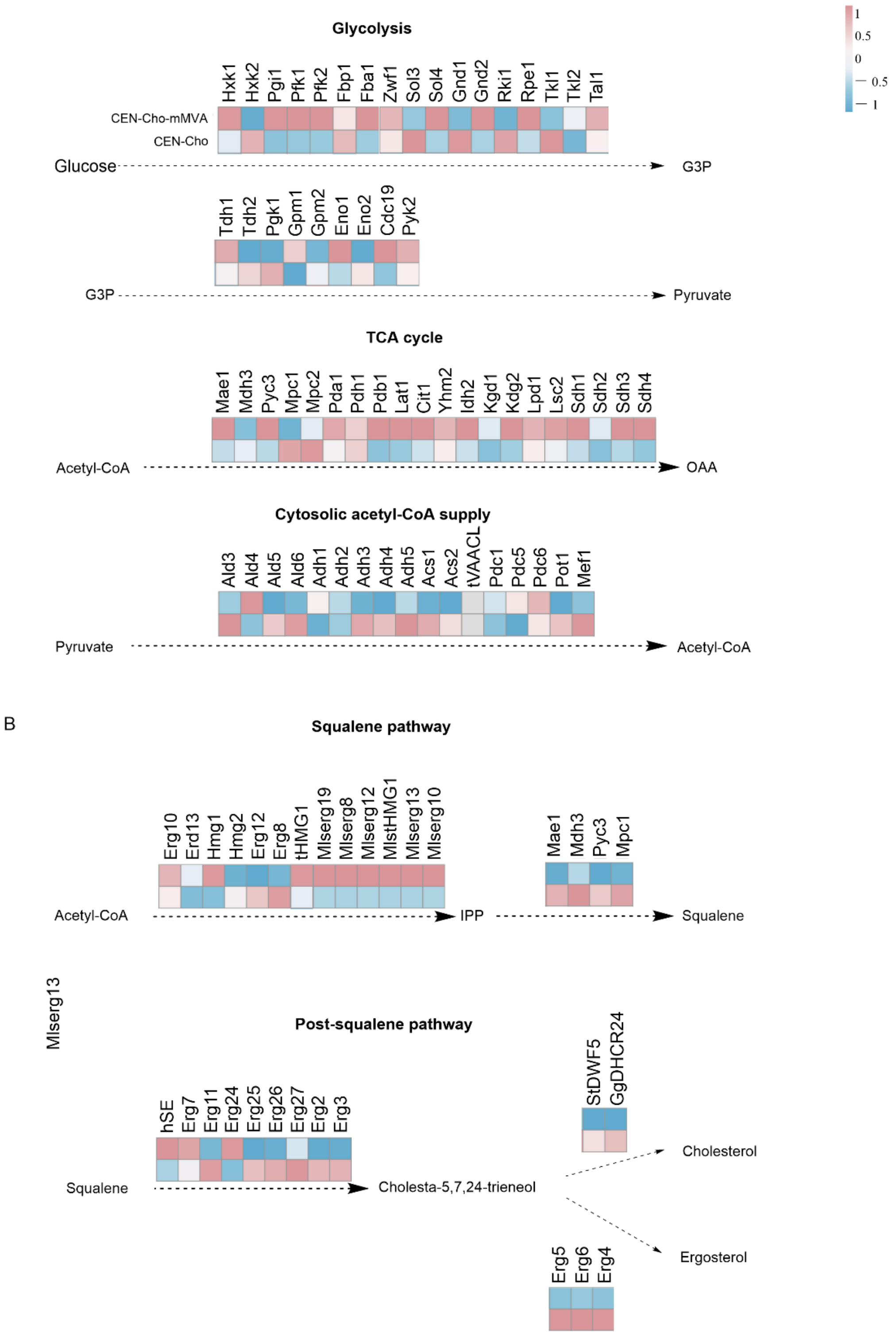

Figure S6. Comparison of CEN-Cho and CEN-Cho-mMVA in the expression of transcripts related to the processes of glycolysis, TCA cycle, and cytosolic acetyl-CoA supply, and the biosynthetic steps up to squalene, cholesterol and ergosterol. The expression data was retrieved from the RNA-sequencing of the yeast cells harvested at the 120 h-time point of Figure S1. Transcript levels were estimated as $\log_2$ (reads per kilobase per million reads (rpkm) +1), and shown in heat map intensity. The expression analysis was performed in triplicate. The heatmap

was drawn using R software (version 4.3.3). The scale from -1 (blue) to +1 (red) represents increased gene expression.

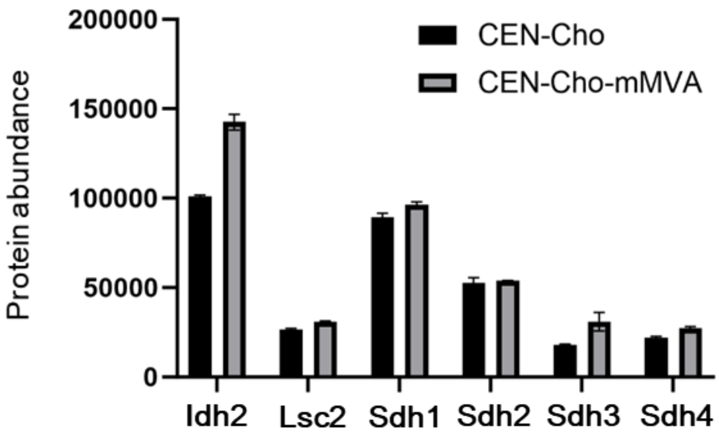

Figure S7. Comparison of the abundance of Idh2, Lsc2, Sdh1, Sdh2, Sdh3 and Sdh4 expressed in the 120 h harvested cells of CEN-Cho and CEN-Cho-mMVA. The analysis was performed in triplicate.

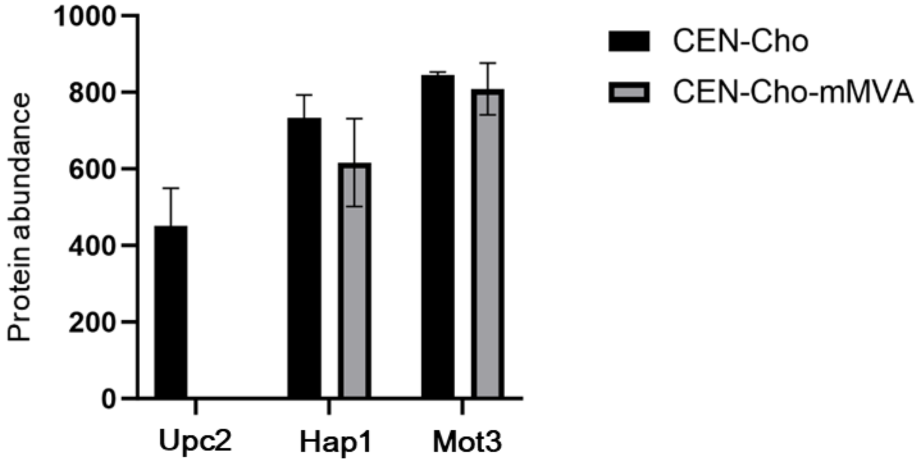

Figure S8. Comparison of the abundance of Upc2, Hap1 and Mot3 expressed in the 120 h harvested cells of CEN-Cho and CEN-Cho-mMVA. The analysis was performed in triplicate.

References

1. Baek, S.; Utomo, J.C.; Lee, J.Y.; Dalal, K.; Yoon, Y.J.; Ro, D.K. The yeast platform engineered for synthetic gRNA-landing pads enables multiple gene integrations by a single gRNA/Cas9 system. *Metab. Eng.* **2021**, *64*, 111–121. https://doi.org/10.1016/j.ymben.2021.01.011.
2. Entian, K.D.; Kötter, P. 25 Yeast Genetic Strain and Plasmid Collections. *Method Microbiol.* **2007**, *36*, 629–666. https://doi.org/10.1016/S0580-9517(06)36025-4.
